# Heat Treatment of Cast and Cold Rolled Al–Yb and Al–Mn–Yb–Zr Alloys

**DOI:** 10.3390/ma14237122

**Published:** 2021-11-23

**Authors:** Veronika Kodetová, Martin Vlach, Lucia Bajtošová, Michal Leibner, Hana Kudrnová, Jaroslav Málek, Vladimír Mára, Miroslav Cieslar, Sebastien Zikmund

**Affiliations:** 1 Faculty of Mathematics and Physics, Charles University, 121 16 Prague, Czech Republic; martin.vlach@mff.cuni.cz (M.V.); lucibajtos@gmail.com (L.B.); mleibner@seznam.cz (M.L.); hana.kudrnova@mff.cuni.cz (H.K.); cieslar@met.mff.cuni.cz (M.C.); sebastienz@seznam.cz (S.Z.); 2 Faculty of Mechanical Engineering, Czech Technical University in Prague, 166 36 Prague, Czech Republic; jardamalek@seznam.cz (J.M.); vladimir.mara@fs.cvut.cz (V.M.)

**Keywords:** Al-based alloys, ytterbium, electrical resistometry, microhardness, Al_3_(Yb,Zr) particles

## Abstract

The microstructure, electrical properties and microhardness of as-cast and cold rolled AlYb and AlMnYbZr alloys were investigated. The addition of Mn, Yb and Zr has a positive influence on grain size. A deformed structure of the grains with no changes of their size was observed after cold rolling. The Al_3_Yb particles coherent with the matrix were observed in the AlYb alloys. The size of the particles was about 20 nm in the initial state; after isochronal treatment up to 540 °C the particles coarsen, and their number density was lower. The deformation has a massive effect on the microhardness behavior until treatment at 390 °C, after which the difference in microhardness changes between as-cast and cold rolled alloys disappeared. Relative resistivity changes show a large decrease in the temperature interval of 330–540 °C which is probably caused by a combination of recovery of dislocations and precipitation of the Al_3_(Yb,Zr) particles. Precipitation hardening was observed between 100 and 450 °C in the AlYb alloy after ageing at 625 °C/24 h and between 330 and 570 °C in the AlMnYbZr alloy after ageing at 625 °C/24 h.

## 1. Introduction

Four of the heaviest rare earth elements (RE = Er, Lu, Tm and Yb) can, similarly to Sc, exhibit an Al_3_RE phase with a stable L1_2_ structure [1,2,3,4]. RE elements are attractive additions to Al-based alloys for many reasons: (a) RE form Al_3_(Sc_1−x_RE_x_) precipitates with the L1_2_ structure, thereby replacing the expensive Sc; (b) diffusivity of RE in Al, which is bigger than of Zr or Ti [1,5]; (c) RE increase the lattice parameter mismatch between α-Al and Al_3_(Sc_1−x_RE_x_) precipitates [6,7]. On the other hand, these elements have low solubilities in Al, even at eutectic temperatures [6,8,9,10]. Additives of Yb in binary Al alloys lead to the formation of L1_2_ structured Al_3_Yb precipitates, which increase hardness and strengthening massively [1,2,11,12]. Tang et al. [3] showed that heat treatment resulted in coherent Al_3_Yb precipitates with a mean radius less than ~11 nm, while those with a mean radius larger than 11 nm were semi-coherent. Several studies (e.g., [12,13,14,15,16,17,18]) observed yield strength increases at ambient temperature in Al alloys due to the addition of Yb and/or Er or an improvement of the creep resistance in the alloys. Moreover, the combined addition of Zr and Yb in Al alloys forms the unique core/shell structured Al_3_(Yb,Zr) which improves recrystallization resistance [4,19]. Compared to Al_3_Zr precipitates, Al_3_(Yb,Zr) precipitates have a higher density and a more homogeneous distribution [4].

The research of the Al–based alloys, with a little addition of Yb, Er and Sc, shows that Yb diffuses faster than Er and Sc in the α-Al matrix [1]. The microhardness results show the highest hardening effect in the Al–0.03 at.% Yb at 200 °C, in the Al–0.03 at.% Er at 250 °C and in the Al–0.12 at.% Sc at 350 °C after isochronal ageing for 2 h time intervals with temperature steps of 50 °C [1]. In comparison to the RE, Zr addition has a very low diffusivity in Al which results decreased rate of Yb precipitation in Al–Yb–Sc(–Zr) alloys [2,20]. Barkov et al. showed that the maximum precipitation hardening effect in Al–Yb–Sc–Zr alloy (during ageing at 300 °C) was mainly caused by Al_3_(Sc,Yb,Zr) dispersoids in a size range of 4–8 nm [21].

Despite several studies on cast Al–Yb(–Zr) alloys (e.g., [1,2,3,4,11,21,22]) there is a lack of a complex characterization of the Al–Yb(–Zr) system, a lack of knowledge on the influence of high temperature treatment and especially of the influence of deformation on precipitation behavior of Al-based alloys with Yb and Zr addition. In this study, we describe the evolution of mechanical and electrical properties of as-cast and cold rolled Al–Yb and Al–Mn–Yb–Zr alloys during isochronal treatment as well as the effect of Mn and Zr addition. In addition, the influence of isothermal ageing at 625 °C for 24 h on the microhardness and microstructure of the alloys was discussed.

## 2. Materials and Methods

Two as-cast alloys (AC) with a chemical composition Al–0.6Yb in wt.% (AlYb) and Al–0.11Mn–0.92Yb–0.12Zr in wt.% (AlMnYbZr) were cold rolled with a reduction thickness of 40% (CR40) and 70% (CR70). The alloys were cast from Al and Mn of commercial purity and master alloys Al–10Yb and Al–9Zr (both in wt.%) melted and stirred in a furnace at 850 °C. The melt was cast into an iron mold in size 110 × 56 × 26 mm^3^. Cold rolling was performed at room temperature (RT). Both alloys were studied in detail up to 600 °C in different types of treatment: (a) isochronal ageing with steps of 30 °C/30 min and/or 60 °C/120 min and (b) isothermal ageing at 625 °C for 24 h (HT). Treatment up to 240 °C was carried out in a stirred silicon oil bath and each step was followed by quenching into liquid nitrogen. Treatment at higher temperatures was performed in an air furnace with Ar protective atmosphere, and this was followed by quenching into water at RT. 

The relative electrical resistivity changes Δ*ρ*/*ρ*_0_ (where *ρ*_0_ is the electrical resistivity value in the initial state) were measured in liquid nitrogen (78 K) using the DC four-point method. The accuracy of absolute resistivity values is limited mainly by the accuracy of the specimen dimensions and was estimated to be better than ~2%. The effect of parasitic thermoelectromotive force was restricted by current reversal. Vickers microhardness (HV0.1) was determined at RT on polished specimens using the Wolpert Wilson Micro Vickers 401MVD (Wilson Instruments, Canton, MA, USA). The samples were stored in liquid nitrogen to protect the development of the microstructure during storage.

The development of microstructure of the alloys was studied using transmission electron microscopy (TEM) and electron diffraction (ED), scanning electron microscopy (SEM) and electron back-scattered diffraction (EBSD). The microscopic observations were carried out on JEOL JSM 7600F (JEOL, Tokyo, Japan), JEOL JEM 2000FX (JEOL, Tokyo, Japan), FEI Quanta 200FEG and MIRA I Schottky FE-SEMH microscopes (TESCAN ORSAY HOLDING, Brno-Kohoutovice, Czech Republic). Energy-dispersive spectroscopy (EDS) was carried out using X-ray BRUKER microanalyser (Bruker AXS, Karlsruhe, Germany).

## 3. Results and Discussion

### 3.1. Initial State of the As-Cast and Cold Rolled Alloys

**Figure 1:**
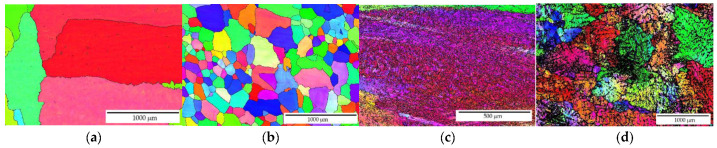
EBSD images of the structure of grains (**a**) AlYb AC, (**b**) AlMnYbZr AC, (**c**) AlYb CR40, (**d**) AlMnYbZr CR70.

The EBSD observation in different locations across the sample shows that the addition of manganese (Mn) and zirconium (Zr) to the AlYb alloy together with higher addition of Yb causes a grain refinement. The grain size of the alloys was in the units of the millimeter range for the AlYb alloy, and approximately tens and/or lower hundreds of μm for the AlMnYbZr alloy (see Figure 1). In the cold rolled materials, the deformation structure was noticed, but without a significant change in the grain size—compare Figure 1a–d.

**Figure 2:**
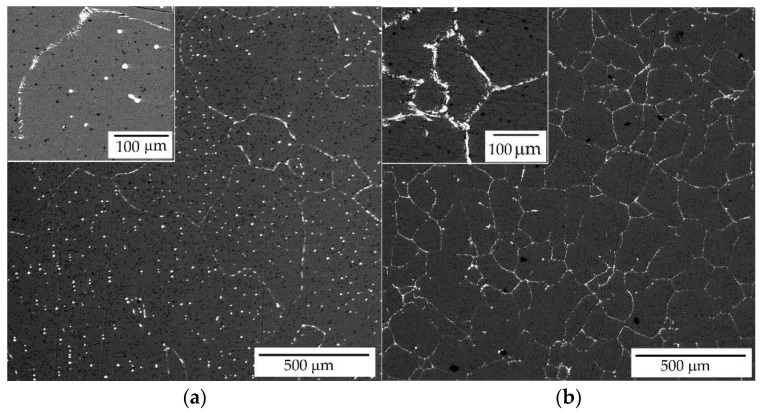
SEM images of the (**a**) AlYb AC and (**b**) AlMnYbZr AC alloys in the initial state. See eutectic phase at the (sub)grains boundary in insets.

SEM images of the AlYb and AlMnYbZr alloys can be seen in Figure 2—the eutectic phase at the (sub)grains boundary was observed in both alloys studied in the initial state. EDS analysis of the eutectic phase was done at several different places across the AlYb and AlMnYbZr samples. Results of AlYb alloy showed that eutectic phase mainly consist of Yb and additionally of Fe. The content of Yb was in range of 0.65–1.20 at.% and the content of Fe was ~0.10 at.% (involving results of measurements of various locations of the eutectic phase). Primary Yb, Fe-rich particles with dimension ~10 μm were observed inside the grains of the AlYb alloy, too (see Figure 2a). EDS analysis of these Yb, Fe-rich particles showed that the content of Yb is in range of 1.14–7.66 at.% and the content of Fe ~0.12 at.%. Results of AlMnYbZr alloys showed that eutectic phase mainly consists of Yb (0.55–3.24 at.%), Mn (0.10–0.22 at.%), Zr (0.18–0.76 at.%) and additionally of Fe ~0.10 at.% and Cu ~0.05 at.%—involving results of measurements of various locations of the eutectic phase.

**Figure 3:**
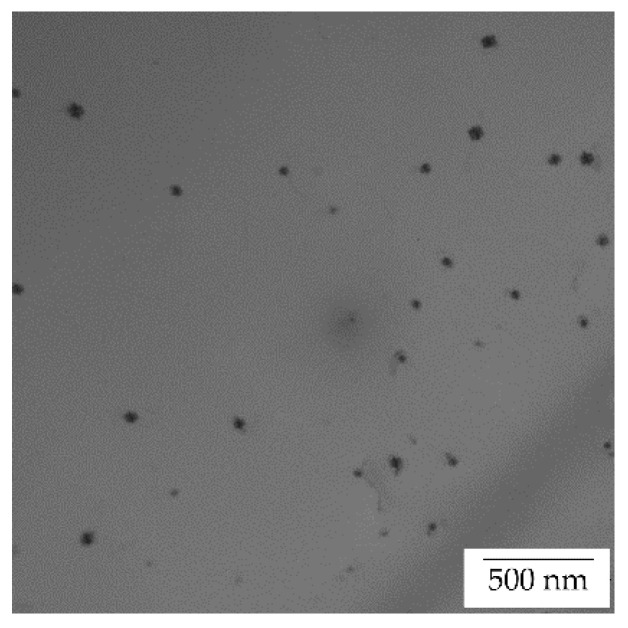
TEM image of the AlYb AC alloy in the initial state—see secondary Al_3_Yb particles.

**Figure 4:**
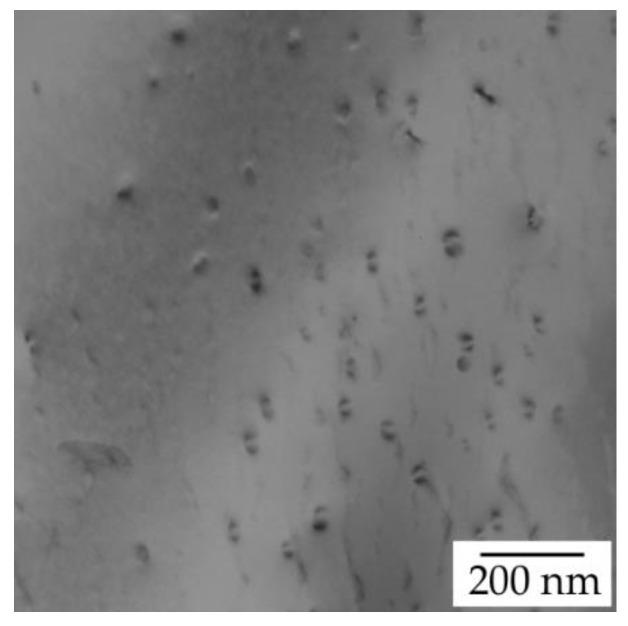
TEM image of the AlMnYbZr AC alloy in the initial state—see secondary Al_3_(Yb,Zr) particles in coffee bean contrast.

TEM of the initial state of the AlYb AC alloy proved particles with dimension ~20 nm—see Figure 3. They are coherent with the Al matrix. Regarding the composition of the AlYb alloy studied and other studies of AlYb alloys (e.g., [1,3,22]), these particles are probably secondary Al_3_Yb particles with the L1_2_ structure. Figure 4 shows the TEM image of the AlMnYbZr AC alloy, where secondary Al_3_(Yb,Zr) particles were observed in the coffee bean contrast. ED proved the L1_2_ structure of these secondary particles. Peng et al. [4] show that the combined addition of Yb and Zr in Al leds to a formation of the coherent Al_3_(Yb,Zr) secondary phase particles with the cubic L1_2_ structure, which provides Al–Zr–Yb alloys with an improved recrystallization resistance. Compared to Al_3_Yb particles observed in the initial state of AlYb alloys, the Al_3_(Yb,Zr) particles (observed in the initial state of the AlMnYbZr alloy) are one order of magnitude smaller and their volume fraction is higher. 

The microhardness values HV0.1 in the initial state of the AlYb and AlMnYbZr alloys are given in Table 1. The difference between the initial microhardness values of AlYb and AlMnYbZr alloys is probably caused by a combination of many factors: the different grain size of the alloys and/or admixture hardening and/or the presence/volume fraction of Al_3_(Yb) and Al_3_(Yb,Zr) secondary particles. The positive influence of cold rolling and/or the addition of Mn and Zr was observed in both studied materials, too. There is no difference (within the accuracy of measurement) in the initial microhardness values of both studied cold rolled alloys with reduction of 40% and 70%. 

### 3.2. Isochronal Treatment of the Alloys

**Figure 5:**
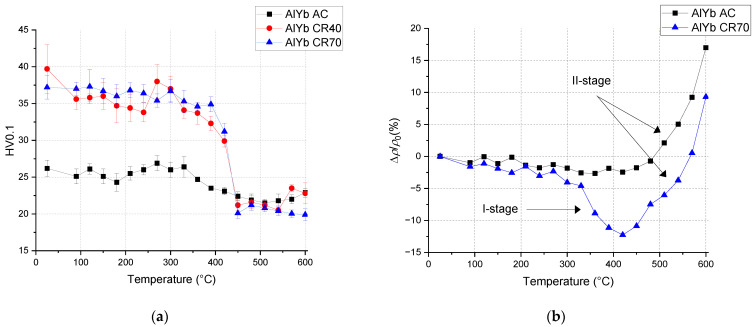
Isochronal ageing curves of the (**a**) microhardness HV0.1 changes and (**b**) relative resistivity changes in AlYb AC and CR alloys.

**Figure 6:**
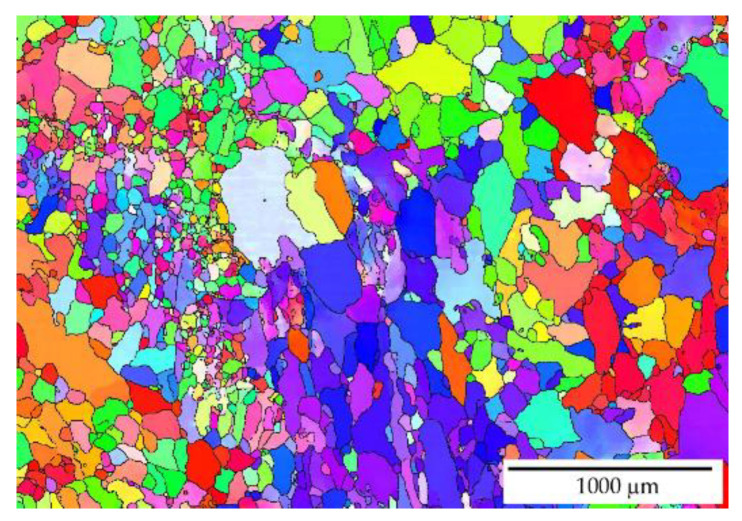
EBSD image of the recrystallized grains in the AlYb AC alloy after ageing at 350 °C for 144 h.

The microhardness HV0.1 and the relative resistivity changes of the as-cast and cold rolled AlYb alloys after isochronal treatment are shown in Figure 5. There are only very slight changes in the microhardness behavior after treatment of the AlYb AC alloy. The results showed almost no difference in HV0.1 between cold rolled alloys in different degrees (40% and 70%) after all steps of treatment. The positive influence of cold rolling on microhardness values is obvious until treatment up to 390 °C; the hardening effect is about 40% here. In the temperature interval 390–450 °C microhardness HV0.1 values of the cold rolled alloys decreases and after treatment higher than 450 °C the microhardness values are almost identical for as-cast and cold rolled state of the AlYb alloys (in the accuracy of measurement). After ageing at 350 °C for 144 h EBSD observation of AlYb alloy proved recrystallization of grains—see Figure 6. The decrease in microhardness in cold rolled AlYb alloys in the 390–450 °C range is probably caused by combination of the recovery of dislocations and/or recrystallization. In the temperature interval 330–420 °C, resistivity of the cold rolled AlYb alloy decreases (labelled as I-stage in Figure 5b) and afterward increases sharply (II-stage). Only the II-stage was observed in the relative resistivity changes curve of the AlYb AC alloy. Relative resistivity changes are more pronounced in cold rolled material. The end of I-stage of relative resistivity decrease is connected to the decrease of microhardness changes in the AlYb alloys studied (Figure 5).

**Figure 7:**
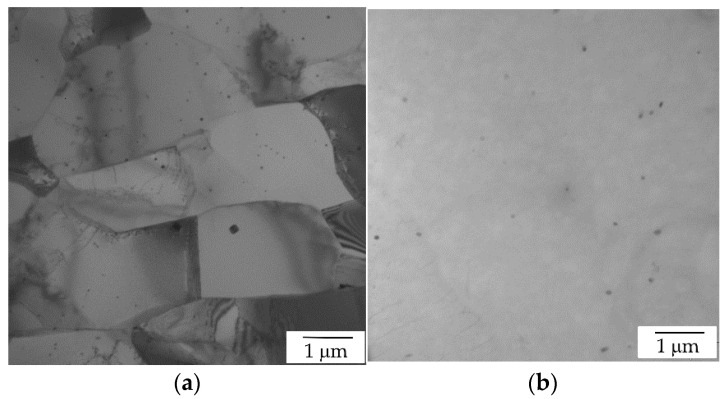
TEM image of the AlYb CR70 alloys after isochronal treatment (**a**) up to 360 °C—see secondary Al_3_Yb particles inside (sub)grains; (**b**) up to 540 °C—see secondary Al_3_Yb particles.

The detailed microstructure analysis of the cold rolled AlYb alloy treated up to 360 °C and up to 540 °C proved the presence of secondary Al_3_Yb particles; see Figure 7 where TEM images of the AlYb CR70 alloys are shown. Compared to the alloy after treatment up to 360 °C, in the alloy after treatment up to 540 °C, particles coarsened and their number density lowered. The I-stage in the relative resistivity changes of AlYb CR70 alloy is probably caused by the combination of additional precipitation of secondary Al_3_Yb particles and/or recovery of dislocation and/or recrystallization. More significant changes in relative resistivity curve in the cold rolled AlYb alloy indicate a positive influence of rolling on the precipitation of the Al_3_Yb particles. These conclusions are consistent with the investigations of Al alloys with other RE additions [23,24]. II-stage in the relative resistivity changes of the AlYb alloys is connected to the dissolution of particles of the Al–Yb system.

**Figure 8:**
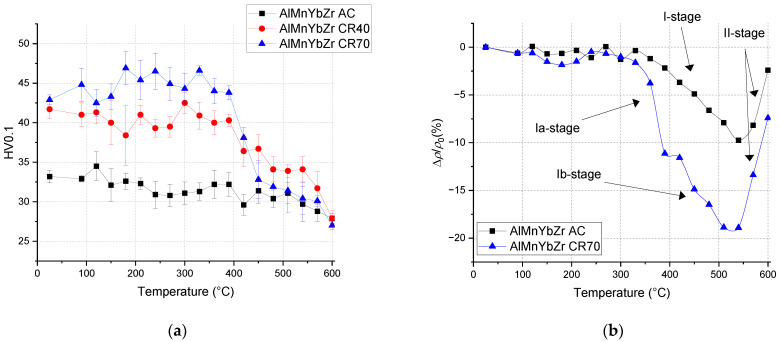
Isochronal ageing curves of the (**a**) microhardness HV0.1 changes and (**b**) relative resistivity changes of the AlMnYbZr AC and CR alloys.

Microhardness HV0.1 and relative resistivity changes of the as-cast and cold rolled AlMnYbZr alloys after isochronal treatment are presented in Figure 8. Insignificant changes were observed in the as-cast AlMnYbZr alloy due to the isochronal treatment. The microhardness values of the cold rolled state with reduction of 40% and 70% are comparable (in the accuracy of measurement). The positive effect of cold rolling on microhardness changes was observed until treatment at 390 °C (hardening is approximately 30%), after that in the temperature interval 390–450 °C microhardness values slowly decrease and reach the same values (in measurement accuracy) as in the AlMnYbZr AC alloy up to the end of the ageing. The trend of the isochronal ageing curves of microhardness and hardening effect caused by cold rolling until 390 °C (ΔHV0.1 ≈ 10) are the same for the AlYb and AlMnYbZr alloys. 

**Figure 9:**
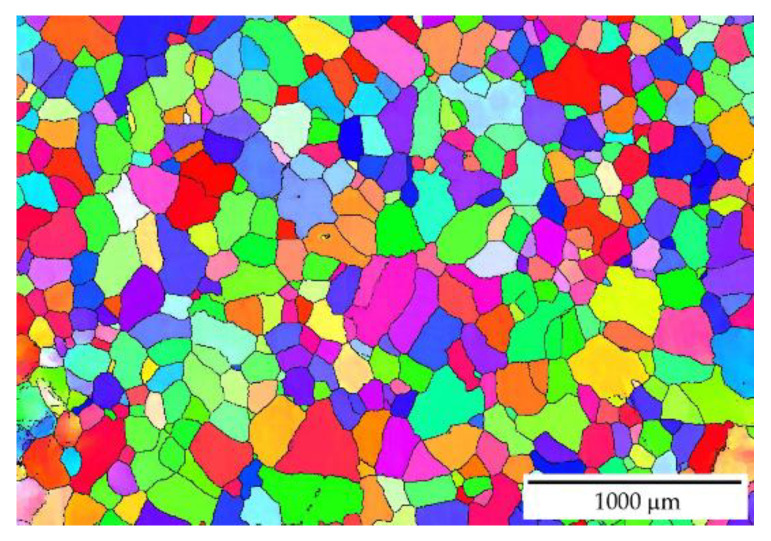
EBSD image after isothermal ageing at 350 °C for 144 h of the AlMnYbZr AC alloy.

Other results [4,23] showed that the addition of Zr to Al alloys forms secondary particles, which strongly inhibit recrystallization. Therefore, we assume that, in contrast to the AlYb alloy, the decrease in microhardness in the cold rolled AlMnYbZr alloys in the temperature interval 390–450 ° C is probably caused only by the recovery of dislocations and/or the recovery of the dislocation structure. This assumption was also verified by EBSD in the as-cast AlMnYbZr alloy, where no change in grain size was observed even after 144 h of ageing at 350 °C in contrast to the AlYb AC alloy after the same ageing procedure—see Figure 1, Figure 6 and Figure 9. In the relative resistivity curves of AlMnYbZr alloys, a decrease of resistivity was observed in the temperature interval 330–540 °C (labelled as I-stage) followed by an increase of resistivity (II-stage). More significant changes and even a double decrease in relative resistivity changes (Ia and Ib-stages) were observed in cold rolled AlMnYbZr alloys.

**Figure 10:**
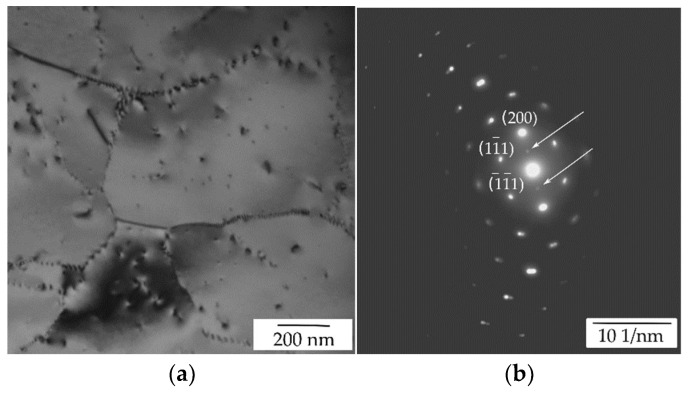
TEM image of the AlMnYbZr AC alloy after isochronal treatment up to 540 °C—see (**a**) secondary Al_3_(YbZr) particles and (**b**) selected area diffraction near [011]_Al_ pole which shows the presence of diffraction spots of the structure L1_2_.

Figure 10 shows the TEM image of the as-cast AlMnYbZr after treatment up to 540 °C. The presence of secondary particles with the L1_2_ structure was proved by ED. With regards to the chemical composition of the alloy, it is very likely to be Al_3_(YbZr) particles. These particles were significantly smaller than in the initial state of the alloy, so isochronal treatment up to 540 °C probably led to their additional precipitation. The resistivity decrease (I-stage) is probably related to the precipitation of these secondary Al_3_(Yb,Zr). According to the literature [1,5,22,23] RE (especially Sc, Zr, Er, Yb) in Al alloys form L1_2_-structured Al_3_(RE) particles coherent with Al matrix with a unique core/shell structure. TEM analysis [4,22,25,26] of these secondary particles showed that two types of particles, corresponding to two different sizes and Zr/Yb ratios, can precipitate in AlZrYb alloys: (a) small dispersoids with low number density and high Zr/Yb ratio and (b) large dispersoids with high number density and low Zr/Yb ratio. Regarding the diffusivities of Yb and Zr in Al alloys [1,5], it was shown that the core/shell structured particles mainly consist of the Al_3_Yb core and the Zr-rich shell [22,26]. It can be seen that cold rolling has a positive influence on the precipitation of Al_3_(Yb,Zr) particles (I-stage) as compared to curves of relative resistivity changes (Figure 8). Moreover, the Ia-stage in cold rolled alloys could be caused by precipitation of Al_3_Yb and the Ib-stage in cold rolled alloys could be caused by wrapping the Yb-rich core by Zr. Treatment above 540 °C probably leads to a dissolution of the Al–(Mn)–Yb–Zr system particles, which corresponds to the increase in the relative resistivity changes (II-stage) and a slight hardening decrease. SEM and EDX analysis show that the Mn in AlMnYbZr alloy probably dissolved in the matrix and thus probably has no influence on ongoing phase transformations.

### 3.3. High Temperature Treatment of the Alloys

The initial microhardness values of the as-cast and high temperature treated AlYb and AlMnYbZr alloys are collected in the Table 2. Isothermal ageing at 625 °C/24 h (labelled as HT) has two effects on microhardness values of the alloys: firstly, this ageing led to the lower values of microhardness in comparison to as-cast AlMnYbZr alloy and, secondly, the difference of microhardness values between AlYb and AlMnYbZr alloys disappeared. 

**Figure 11:**
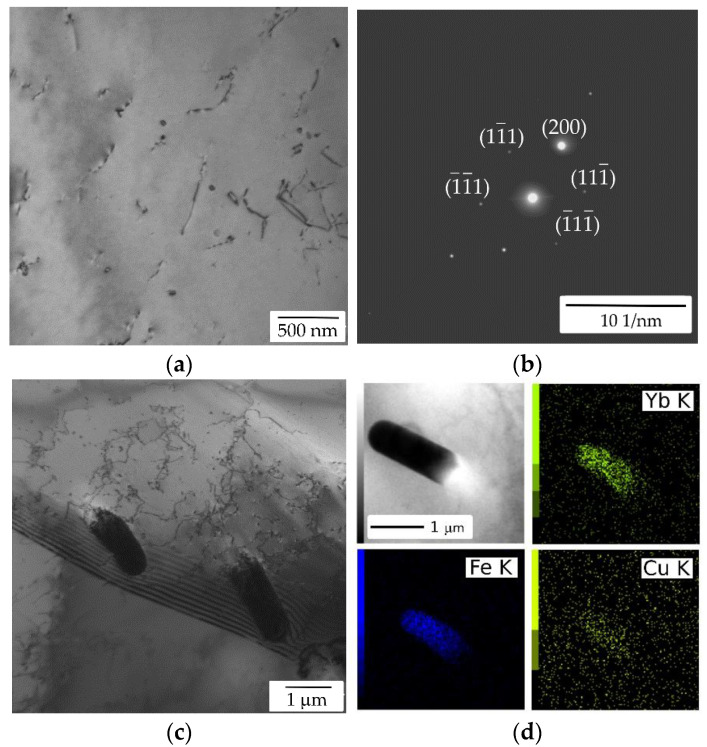
TEM image of the AlMnYbZr HT alloy—see (**a**) dislocations, (**b**) selected area diffraction near [011]_Al_ pole, (**c**) particles in grain boundary and (**d**) EDS map of the particle.

Figure 11 shows the TEM (and ED) image of the AlMnYbZr alloys after high temperature isothermal ageing at 625 °C/24 h. Particles about units of μm large were observed only in the grain boundaries in the AlMnYbZr HT alloy—see Figure 11c. EDS analysis proved that these particles are rich mainly in Fe, Yb, and Cu, see EDS map in Figure 11d. No particles and/or phases were observed neither by TEM nor by ED in the AlYb alloy. Comparing TEM images and results in the initial states of as-cast alloys (Figure 3 and Figure 4—where Al_3_Yb(Zr) particles are seen) and high temperature treated alloys (Figure 11) one can conclude, that this ageing led to the dissolution of Al_3_Yb(Zr) particles. The difference in microhardness values of as-cast AlMnYbZr and high temperature treated alloys is probably caused by the presence of secondary Al_3_(YbZr) particles in the as-cast state.

**Figure 12:**
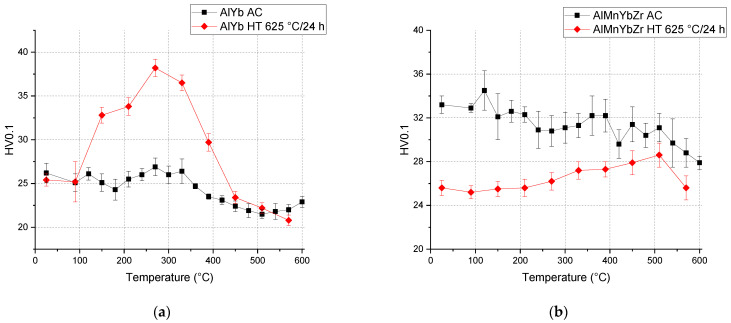
Isochronal ageing curves of microhardnes changes HV0.1 for the (**a**) AlYb AC and HT alloys and (**b**) AlMnYbZr AC and HT alloys. Isochronnal treatment of AC alloys was done in steps of 30 °C/30 min and of HT alloys in steps of 60 °C/120 min.

The microhardness changes HV0.1 of the high temperature treated AlYb and AlMnYbZr alloys during isochronal treatment in steps 60 °C/120 min are presented in Figure 12. The highest hardening effect was observed in the temperature range 100–450 °C in the AlYb alloys. Except for this region, the microhardness values are almost similar (in the accuracy of measurement) to the microhardness values of the as-cast alloy. It seems that the hardening effect in the AlYb HT alloy has a double peak with maxima at ~150 °C (ΔHV0.1 ≈ 8) and ~270 °C (ΔHV0.1 ≈ 13). The situation in the AlMnYbZr alloys is different—the microhardness values after high temperature ageing are lower than those of the AlMnYbZr AC alloy after all steps of isochronal treatment. The fine hardening effect in the temperature range of 330–570 °C with a maximum at 510 °C was observed for the high temperature treated AlMnYbZr alloy.

**Figure 13:**
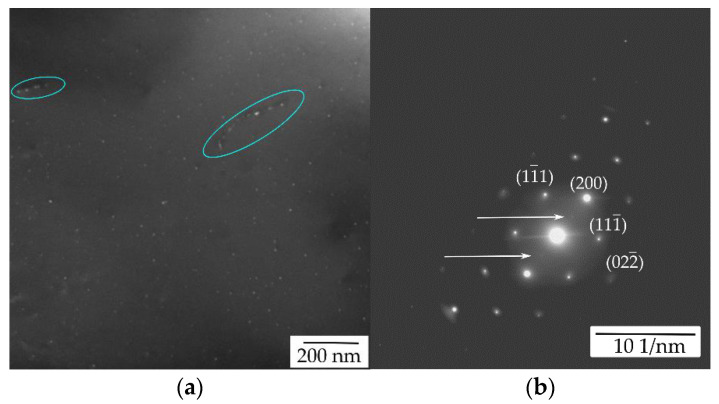
TEM image of the AlYb HT alloy after isochronal treatment up to 330 °C—see (**a**) secondary Al_3_Yb particles, some of them precipitated along dislocations and (**b**) selected area diffraction near [011]_Al_ pole with spots from the L1_2_ structure.

**Figure 14:**
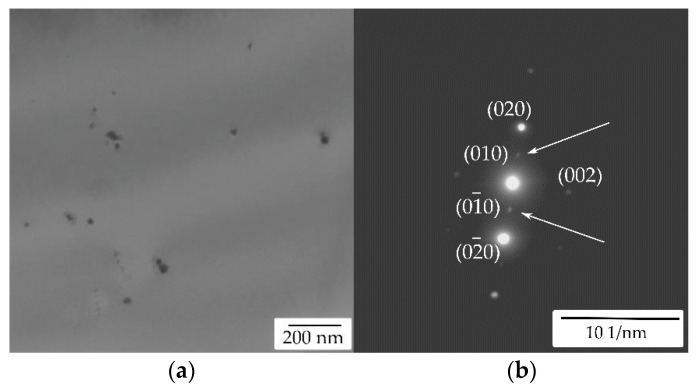
TEM image of the AlYb HT alloy after isochronal treatment up to 510 °C—see (**a**) secondary Al_3_Yb particles with different sizes and (**b**) selected area diffraction near [100]_Al_ pole with spots from the L1_2_ structure.

Detailed TEM and ED analysis of the high temperature treated AlYb alloy after isochronal treatment up to 330 °C proved presence of fine secondary Al_3_Yb particles with the L1_2_ structure, some of them precipitated along dislocations—see Figure 13, where particles precipitated along dislocations are marked in blue ovals. TEM analysis showed that these secondary Al_3_Yb particles coarsened and the number density decreases after treatment up to 510 °C (see Figure 14). The double hardening peak observed in the microhardness curves of the AlYb HT alloy in the temperature interval of 100–450 °C is probably caused by massive precipitation of secondary Al_3_Yb particles. These results agree with studies of Van Dalen et al. and Zhang et al. [1,26]. They showed that microhardness of an Al0.03Yb alloy (in at.%) aged at 625 °C for 72 h increased in a double peak after isochronal ageing in the temperature range of ~50–350 °C. The investigation proved that this hardening is caused by homogenous and heterogeneous precipitations of secondary Al_3_Yb particles; some of them precipitate (heterogeneously) along dislocations [1]. Low temperature ageing promotes homogeneous nucleation and/or homogeneous distribution of the Al_3_RE precipitates, furthermore, at higher ageing temperatures heterogeneous nucleation dominates [27]. According to Van Dalen at al. heterogeneous precipitates (in Al0.03 at.% Yb alloy) have a mean radius ≈10 nm, which is larger than those that are homogeneously distributed (≈3.8 nm) [1]. On the base of literature (e.g., [1,2,11]) and TEM observation we assume, that the first hardening peak (with maximum at ~150 °C) observed in AlYb HT alloy studied in this work is connected with homogeneous precipitation of the Al_3_Yb particles and the other (with maximum at ~270 °C) is connected with heterogeneous nucleation of the Al_3_Yb.

**Figure 15:**
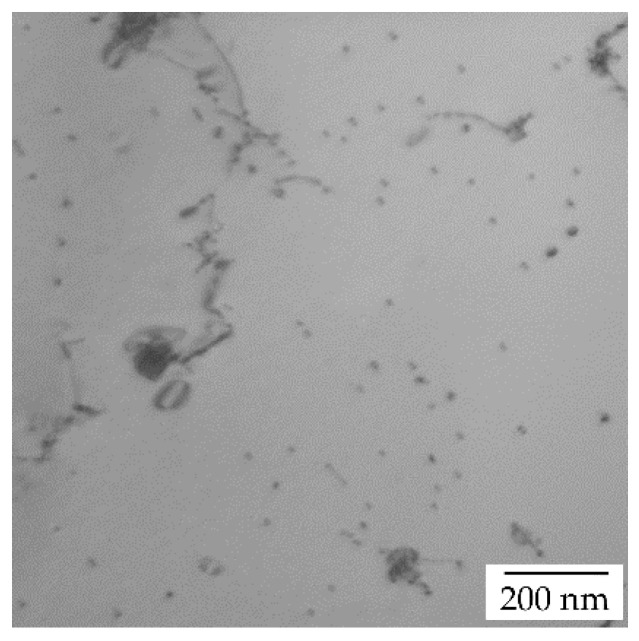
TEM image of the AlMnYbZr HT alloy after isochronal treatment up to 510 °C—see secondary Al_3_(Yb,Zr) particles.

In the high temperature treated AlMnYbZr alloy after isochronal treatment up to 510 °C (state with the highest hardening effect) TEM and ED revealed presence of secondary Al_3_(Yb,Zr) particles—see Figure 15. Moreover, Fe, Yb and Cu-rich grain boundary phases (observed in the initial state of the HT alloy) were dissolved after isochronal treatment up to 510 °C. It seems that the volume fraction of the secondary Al_3_(Yb,Zr) is lower for AlMnYbZr HT alloy after isochronal treatment up to 510 °C than for the initial state of the AlMnYbZr AC alloy, compare Figure 4 and Figure 15. Lower microhardness value in the AlMnYbZr HT alloy after treatment up to 510 °C than in the initial state of the as-cast alloy is probably connected to the lower volume fraction of these secondary Al_3_(Yb,Zr) particles.

## 4. Conclusions

The results of the as-cast, cold rolled and high temperature treated AlYb and AlMnYbZr alloys obtained by electrical resistometry, microhardness and microstructure observations follow these key results:

(a) The addition of manganese (Mn) and zirconium (Zr) to the AlYb alloy together with higher addition of Yb cause a grain refinement. The grain size of the alloys was approximately units of mm for the AlYb alloy and about tens of μm for the AlMnYbZr alloy. In the cold rolled materials, the deformation texture was noticed, but without a significant change in the grain size.
(b) The eutectic phase at (sub)grains boundaries was observed in both studied alloys in the initial as-cast state. This phase mainly consists of Yb and Fe in the AlYb alloy and mainly of Yb, Mn, Zr and additionally of Cu and Fe in the AlMnYbZr alloy.
(c) TEM in the initial state of the AlYb AC and AlMnYbZr AC alloy proved secondary Al_3_Yb and Al_3_(Yb,Zr) phase particles with the L1_2_ structure. Massive precipitation and/or coarsening of these secondary particles were observed in the alloys after treatment up to 330 °C and 540 °C.
(d) The difference between the initial values of microhardness in AlYb and AlMnYbZr alloys is probably caused by a combination of the different grain size in the alloys and/or admixture hardening and/or the presence/volume fraction of Al_3_Yb and Al_3_(Yb,Zr) secondary particles. The positive influence of cold rolling was observed in both studied materials up to treatment at 390 °C. The decrease of microhardness in the cold rolled alloys in the range 390–450 °C is probably caused by recovery of dislocations and/or recrystallization in the AlYb alloy and by recovery of dislocations in the AlMnYbZr alloy. Almost no difference in microhardness values between cold rolled materials in different degrees (40% and 70%) after all steps of treatment was observed.
(e) The decrease of relative resistivity (up to 420 °C in the as-cast AlYb alloy and up to 510 °C in the as-cast AlMnYbZr alloy) followed by an increase in resistivity were observed. More significant changes and even a double decrease in relative resistivity changes were observed in the cold rolled AlMnYbZr alloys. The decrease in the relative resistivity changes is likely caused by a combination of additional precipitation of secondary Al_3_Yb particles and/or recrystallization in the AlYb alloy. A double decrease in the cold rolled AlMnYbZr alloy is caused by precipitation of Al_3_Yb and by wrapping the Yb-rich core by Zr. The increase in the relative resistivity changes of the AlYb and AlMnYbZr alloys is connected to the dissolution of particles of the Al–Yb and Al–Yb–Zr system. Small addition of Mn (0.11 wt.%) has no influence of phase transformation.
(f) No particles were observed in the AlYb alloy in contrast to AlMnYbZr alloy, where Fe, Yb and Cu-rich particles in size of μm were observed in grains boundaries after high temperature ageing at 625 °C/24 h.
(g) The hardening effect with a double peak was observed in the temperature range 100–450 °C in the AlYb alloy after the high temperature ageing 625 °C/24 h. The first hardening peak (with maximum at ~150 °C) is connected to homogeneous precipitation of Al_3_Yb particles and the other (with maximum at ~270 °C) is connected to heterogeneous nucleation of the Al_3_Yb. A slight hardening effect in the temperature range of 330–570 °C with a maximum at 510 °C was observed after the high temperature ageing of the AlMnYbZr alloy, connected to the precipitation of Al_3_(Yb,Zr) particles.

## Figures and Tables

**Table 1:** Initial values of microhardness HV0.1 for the AlYb and AlMnYbZr as-cast (AC) and cold rolled alloys with reduction of 40% (CR40) and 70% (CR70).

Alloy	HV 0.1
AlYb AC	26 ± 1
AlYb CR40	40 ± 3
AlYb CR70	37 ± 2
AlMnYbZr AC	33 ± 1
AlMnYbZr CR40	42 ± 1
AlMnYbZr CR70	43 ± 1

**Table 2:** Initial values of microhardness HV0.1 for the AlYb and AlMnYbZr alloys in the as-cast state (AC) and after high temperature ageing at 625 °C for 24 h (HT).

Alloy	HV 0.1
AlYb AC	26 ± 1
AlYb HT	25 ± 1
AlMnYbZr AC	33 ± 1
AlMnYbZr HT	26 ± 1

## Data Availability

The data used to support the findings of this study are available from the corresponding author upon request.
